# Inhibition of autophagy sensitizes cancer cells to Photofrin-based photodynamic therapy

**DOI:** 10.1186/s12885-018-4126-y

**Published:** 2018-02-20

**Authors:** Antoni Domagala, Joanna Stachura, Magdalena Gabrysiak, Angelika Muchowicz, Radoslaw Zagozdzon, Jakub Golab, Malgorzata Firczuk

**Affiliations:** 1 Department of Immunology, Medical University of Warsaw, 1A Banacha Str., F building, 02-097 Warsaw, Poland; 2 Postgraduate School of Molecular Medicine, Warsaw, Poland; 3 Immunity&Cancer Laboratory, The Francis Crick Institute, London, UK; 4 Department of Clinical Immunology, Transplantation Institute, Medical University of Warsaw, Warsaw, Poland; 5 Department of Bioinformatics, Institute of Biochemistry and Biophysics, Polish Academy of Sciences, Warsaw, Poland; 6 Centre for Preclinical Research and Technology, Medical University of Warsaw, Warsaw, Poland

**Keywords:** Autophagy, Photodynamic therapy, Photofrin, ATG5, CRISR/Cas-9

## Abstract

**Background:**

Accumulating evidence suggest that autophagy plays a pivotal role in various anticancer therapies, including photodynamic therapy (PDT), acting as a pro-death or pro-survival mechanism in a context-dependent manner. Therefore, we aimed to determine the role of autophagy in Photofrin-based PDT.

**Methods:**

In vitro cytotoxic/cytostatic effects of PDT were evaluated with crystal violet cell viability assay. Autophagy induction was analyzed by immunoblotting and immunofluorescence using anti-LC3 antibody. Autophagy was inhibited by shRNA-mediated ATG5 knockdown or CRISPR/Cas9-mediated ATG5 knockout. Apoptosis was assessed by flow cytometry analysis of propidium iodide and anexin V-positive cells as well as by detection of cleaved PARP and caspase 3 proteins using immunoblotting. Protein carbonylation was evaluated by the 2,4-dinitrophenylhydrazine (DNPH) method.

**Results:**

Photofrin-PDT leads to robust autophagy induction in two cancer cell lines, Hela and MCF-7. shRNA-mediated knockdown of ATG5 only partially blocks autophagic response and only marginally affects the sensitivity of Hela and MCF-7 cells to PDT. ATG5 knockout in HeLa cell line utilizing CRISPR/Cas9 genome editing results in increased PDT-mediated cytotoxicity, which is accompanied by an enhanced apoptotic response and increased accumulation of carbonylated proteins.

**Conclusions:**

Altogether, these observations imply that autophagy contributes to Photofrin-PDT resistance by enabling clearance of carbonylated and other damaged proteins. Therefore, autophagy inhibition may serve as a strategy to improve PDT efficacy.

## Background

Autophagy is an evolutionary conserved catabolic process by which damaged organelles or long-lived proteins are targeted for lysosomal degradation [1, 2]. Although autophagy is constitutively active at basal rate, it is predominantly induced by stressful stimuli disturbing cellular homeostasis [3]. In general, autophagy is considered as a cytoprotective mechanism facilitating survival under unfavorable conditions, yet, it can also facilitate cell death [4].

Autophagy involves sequestration of cytoplasmic constituents into double-membraned vesicles, termed autophagosomes, which are subsequently delivered to lysosome for their degradation [5]. During autophagy, a cytosolic protein, LC3-I, is converted to its lipidated form LC3-II, which is recruited to autophagosomal membrane. The whole pathway is orchestrated by two ubiquitin-like conjugation systems, which employ autophagy-related genes (ATG). Several ATG genes are critical for the conversion of LC3 including ATG5 [6, 7]. Accumulating evidence indicates that autophagy is involved in tumor formation and progression, as well as response to anticancer therapies [8]. However, the exact role of this process is still controversial [9, 10]. The prevailing current views indicate that autophagy can either promote or inhibit cell proliferation in a context dependent manner [11].

Photodynamic therapy (PDT) is a clinically approved and well-established anticancer therapy [12]. The unique mechanism of action of PDT is based on the administration of photosensitizing agent, which is subsequently activated via light exposure to produce reactive oxygen species (ROS) [13, 14]. ROS are responsible for photodamage of proteins and macromolecules, which subsequently leads to the destruction of malignant cells [15]. It has been also described that photodamage can result in autophagy induction [16, 17].

There are numerous studies investigating autophagy in the context of photodynamic therapy. However, as it has been summarized in a recent review [18], PDT-induced autophagy contributes to cell death and survival in roughly the same number of cases. This highlights the need to further study the role of PDT-induced autophagy as this process has not been fully elucidated so far. Significance of autophagic pathway in photodynamic therapy is complex and depends on numerous factors, including cell type, light dose, access to oxygen, as well as the type of photosensitizer and its subcellular localization. Previous reports evaluating autophagy in the context of photodynamic therapy involved mainly photosensitizers which accumulate in mitochondria [16, 19] or endoplasmic reticulum [20, 21]. However, little is known whether autophagy is triggered by PDT with the use of Photofrin, which localizes mainly in cell membranes [22]. Moreover, one report suggests that Photofrin alone, without light activation, can act as an autophagy inhibitor [23]. Thus, we aimed to investigate whether Photofrin-PDT triggers autophagy and whether autophagic pathway contributes to increased sensitivity or resistance of cancer cells towards photodynamic therapy.

## Methods

### Cell culture

Human cervical cancer cell line - Hela (ATCC® CRM­CCL­2™) was purchased from American Type Culture Collection. Breast cancer cell line - MCF-7 (86012803) was purchased from European Collection of Cell Culture. Cell cultures were maintained under standard conditions in a 5% CO_2_ humidified incubator at 37 °C in DMEM (HeLa) or RPMI (MCF-7) supplemented with 10% heat-inactivated fetal bovine serum and penicillin-streptomycin solution.

### Reagents and chemicals

Photofrin, used as a photosensitizer in the study, was dissolved in PBS (stock concentration 0.5 mg/ml), aliquoted and stored at − 80 °C. All other chemicals were purchased from Sigma-Aldrich, unless stated otherwise.

### In-vitro photodynamic therapy

Cells were dispensed into 35-mm or 60-mm plates and allowed to attach overnight. After additional 24-h incubation with 10 μg/ml Photofrin, culture medium was replaced by PBS and cells were illuminated with 100 W sodium lamp (Philips) through a red filter. This was followed by additional 24-h incubation in fresh medium.

### Cell viability assay

Cytostatic/cytotoxic effects of PDT were determined using crystal violet staining. 24 h after illumination, cells were washed with PBS and stained with 0.5% crystal violet in 20% ethanol for 15 min. Plates were washed extensively with tap water and cells were lysed with 2% SDS. The absorbance was measured at 595 nm using microplate reader (ASYS, UVM 340, Biochrom, Berlin, Germany). The relative viability was calculated as follows: relative viability = [(experimental absorbance − background absorbance)/(untreated control absorbance − background absorbance)] × 100%.

### Annexin/PI staining

Cells were detached with TRYPLE-express (Thermo Scientific), washed with PBS and resuspended in binding buffer, followed by Annexin-V-FITC and PI staining for 15 min. The percentage of annexin- and PI- positive cells was determined using flow cytometer (Accuri, Becton Dickinson, San Jose, USA).

### Western blot

At indicated times after PDT, cells were lysed in a lysis buffer (50 mM HEPES pH 7.4, 1% Triton X-100, 150 mM NaCl, 10% glycerol, 5 mM EDTA) supplemented with protease inhibitors (Roche, Mannheim, Germany). After measuring protein concentration using Protein Assay (Bio-Rad, Hercules, CA, USA), equal amounts of proteins were separated by SDS-PAGE electrophoresis and transferred to nitrocellulose membrane (Schleicher and Schuell BioScience, Dassel, Germany). Membranes were incubated with following primary antibodies: LC3–2775, ATG5–9980, caspase 3–9665, PARP-9542 (Cell Signalling, Beverly, MA, USA) according to the manufacturer’s recommendations. After TBST washing, membranes were probed with HRP-linked secondary antibodies (Cell Signaling), developed with self-made ECL (50 mM Tris-HCl pH 8.5, 0.2 mM coumaric acid, 1.25 mM luminol, 0.006% hydrogen peroxide) or Supersignal West Femto ECL (Thermo Scientific, Waltham, MA, USA) and visualized with Stella 8300 bio-imager (Raytest, Straubenhardt, Germany) or ChemiDoc Touch (Bio-Rad). To ensure equal protein loading, the membranes were reprobed with anti-β-actin antibody (Sigma-Aldrich, Saint Louis, MO, USA: A2228).

### Protein carbonylation

Protein carbonylation was determined by the 2,4-dinitrophenylhydrazine (DNPH) method as described previously [24].

### Fluorescence microscopy

Fluorescent microscopy analysis was performed as described previously [25]. Briefly, cells were seeded on poly-L-lysine-coated glass slides. At indicated time post PDT, cells were fixed with ice-cold methanol and blocked with 2% BSA and 0.5% Triton-X. Slides were incubated overnight with rabbit anti-LC3B antibody (Cell Signalling, 2775) at 4 °C. After PBS washing, cells were stained with Alexa Fluor-488 anti-rabbit antibody (Invitrogen, A21206) and mounted using Vectashield with DAPI (Vector Laboratories, Burlingame, CA, USA). Images were captured using Axio Scan Z1 (Carl Zeiss, Jena, Germany) powered by Zen 2 software.

### ATG5 downregulation using shRNA

HeLa and MCF-7 cells were infected with lentiviral particles (Sigma-Aldrich, St. Louis, MO) according to the manufacturer’s instructions. The following targeting sequences were used: specific shRNA complementary to ATG5 (MISSION shRNA TRCN0000151474) and scrambled (non-targeting) shRNA (SHC002V). After transduction, puromycin (2 μg/ml) was added to culture medium as a selection antibiotic to obtain stable cell lines.

### ATG5 downregulation using CRISPR/Cas9

The lentiCRISPR v2 vector (Addgene, Cambridge, MA, USA:52961) was digested with BsmBI (Fast Digest-Thermo Scientific) and ligated to annealed and phosphorylated sgRNA oligonucleotides containing following sequences: sgATG5–5’-TTCCATGAGTTTCCGATTGA-3′ and sgGFP-5’-GGGCGAGGAGCTGTTCACCG-3′ (sgRNA targeting GFP used as a negative control). For lentivirus production, HEK-293 T cells were co-transfected with lentiCRISPRv2 vector together with psPAX2 and pMD2.G plasmids using Lipofectamine™ 2000 (Thermo Scientific) according to manufacturer’s instruction. After 72 h, lentivirus-containing medium was filtered through 0.45 μM filter and added to HeLa cells. After puromycin selection (2 μg/ml) the surviving cells were seeded into 96-well plate at a density of 0.5 cell/well to grow single-cell clones. To confirm the occurrence of the genetic modification, DNA was extracted using Cell Culture DNA Purification kit (EURX, Gdansk, Poland) according to manufacturer’s recommendations. The genomic region surrounding the target site of guide sequence was PCR amplified with Phusion® polymerase (NEB, Ipswich, MA, USA), gel purified and subsequently sequenced using Sanger method with the following pair of primers 5’-AAATGGCTGTGCGAATATCTAGG-3′ and 5’-ATTTCAGTGCGGTATCTGACTTG-3′.

### Statistical analysis

Data are expressed as means ± S.D. (represented by error bars) and significance was determined with Student’s *t*-test. Analyses were performed using GraphPad Prism 6 software (La Jolla, CA, USA). *P*-values * < 0.05 and ** < 0.001 were considered as significant.

## Results

### Induction of autophagy by Photofrin-PDT

**Fig. 1:**
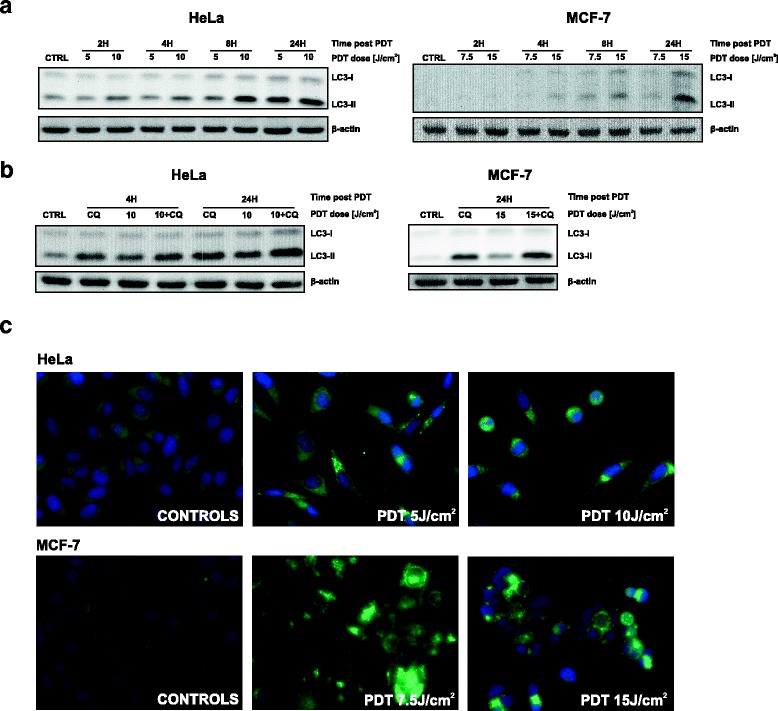
Photofrin-PDT induces autophagy. **a** HeLa (left panel) and MCF-7 (right panel) cells were incubated with Photofrin for 24 h before exposure to different light fluencies. Whole-cell lysates were collected at indicated time points after PDT and analyzed by Western blotting for LC3 and β-actin (loading control) expression. **b** The experiment was performed as in (a), but, for indicated samples, 10 μM chloroquine (CQ) was added to the culture medium after PDT. **c** 16 h after irradiations, the cells were fixed and stained with anti-LC3 antibody to visualize autophagosomes by immunofluorescence

To test if autophagy is triggered upon Photofrin-PDT, HeLa and MCF-7 cells were subjected to the PDT and the cell lysates were collected at various time points post PDT. Western blot analysis revealed that Photofrin-based PDT leads to a conversion of cytosolic LC3-I to its lipidated, membrane-bound form-LC3-II (Fig. 1a), a specific and characteristic marker of autophagy [26]. We have also observed that LC3 is lipidated in a time- and dose-dependent manner.

To rule out the possibility that the accumulation of LC3-II reflects a block in the later stages of autophagy, such as impaired autophagosome degradation rather than autophagy induction, we performed LC3 turnover assay [27]. Co-treatment with autophagy inhibitor chloroquine, which is known to inhibit lysosomal degradation of autophagosomes, led to more pronounced LC3-II accumulation than photodynamic therapy alone (Fig. 1b). These observations indicate that LC3-II accumulation reflects increased autophagy induction rather than impaired autophagic flux.

To confirm autophagy induction with an alternative approach, the autophagosomes were visualized by means of immunofluorescence microscopy. Hela and MCF-7 cells were stained with anti-LC3 antibody 24 h post PDT. As presented in Fig. 1c, PDT led to a substantial increase in the number of punctate structures representing autophagosomal vesicles.

Altogether, our results demonstrate that Photofrin-based PDT leads to a strong autophagy induction, which was confirmed by enhanced LC3 processing and increased autophagosome formation.

### shRNA-mediated ATG5 downregulation moderately affects autophagy induction by PDT and PDT efficacy

**Fig. 2:**
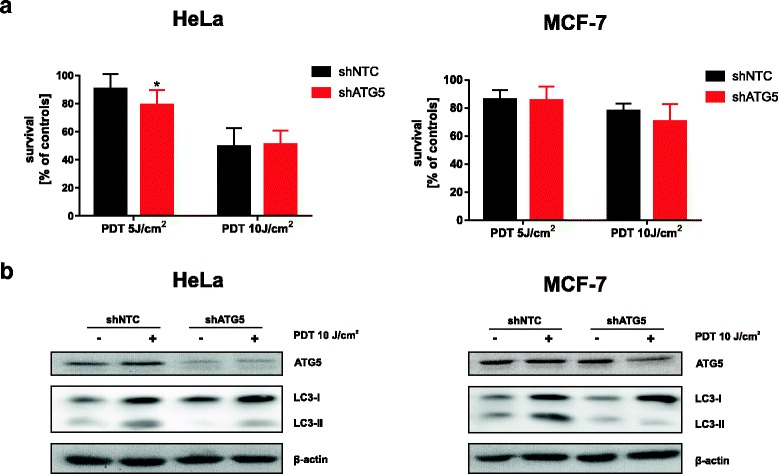
ShRNA-mediated ATG5 downregulation moderately affects autophagy and PDT efficacy. **a** HeLa and MCF-7 cells were infected with lentiviral particles containing shRNA targeting ATG5 (shATG5) or scrambled shRNA (shNTC) and subsequently incubated with puromycin to isolate stable cell lines. The stable cell lines were subjected to in-vitro PDT and cell survival was determined 24 h post-PDT by crystal violet staining. The bars represent survival in each experimental versus its own untreated control. Data show the mean values of 2 independent experiments ± S.D. (represented by error bars), and *P < 0.05 (Student’s *t*-test) (**b**) Whole-cell lysates from shATG5 and shNTC cell lines were collected 24-h post PDT and ATG5 and LC3 expression was evaluated by Western blotting. β-actin expression was assessed as protein loading control

One of the methods to study the role of autophagy involves genetic inhibition of the genes associated with this pathway. It has been demonstrated that ATG5 forms the complex with ATG12, which is indispensable in autophagosome formation and LC3 lipidation [28, 29]. Therefore, we have decided to downregulate ATG5 via shRNA approach to assess how this modification would influence the sensitivity of cancer cells to photodynamic therapy. As revealed in immunoblotting, ATG5 expression, as well as the conversion of LC3-I to LC3-II, was reduced in HeLa and MCF-7 cells transfected with lentiviral particles encoding ATG5-specific shRNA (shATG5) in comparison to mock cells transfected with scrambled shRNA (shNTC, non-targeting controls) (Fig. 2b). Despite this, knockdown mediated by shRNA only moderately influenced the cytostatic/cytotoxic effects of PDT (Fig. 2a). shATG5 HeLa cells were slightly more susceptible to low-dose PDT than mock cells (shNTC), but there were no significant differences at higher PDT doses. In a similar experimental setting, MCF-7 cells with or without ATG5 were equally sensitive to PDT.

### Construction and characterization of HeLa cells with CRISPR/Cas9-mediated genomic knockout of ATG5

**Fig. 3:**
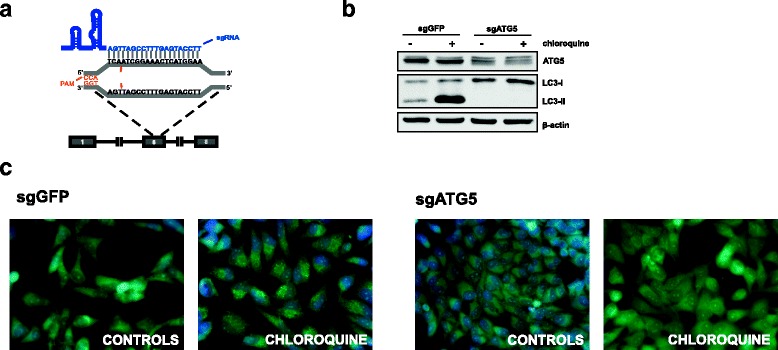
Construction and characterization of HeLa cells with CRISPR/Cas9-mediated genomic KO of ATG5. **a** Schematic diagram showing ATG5 gene and the sgRNA target site at exon 6. Cas-9-mediated double-strand break near the PAM sequence are indicated by arrows. **b** HeLa-sgGFP and HeLa-sgATG5 cells were incubated for 24 h in the presence or absence of 10 μM chloroquine (CQ) to block lysosomal degradation. Whole-cell lysates were collected and ATG5 and LC3 expression was analyzed by Western blotting. **c** To confirm impaired autophagosome formation, HeLa-sgGFP and HeLa-sgATG5 cells were incubated as in (b) and subsequently stained with anti-LC3 antibody to visualize autophagosomes by immunofluorescence microscopy

The shRNA approach to downregulate gene expression turned out to be moderately effective (Fig. 2b) and might be insufficient to block autophagic flux, which is consistent with the published report [30]. Therefore, we utilized CRISPR-Cas9 genome editing technique to generate ATG5 knockout HeLa cell line. Single guide RNA (sgRNA) targeting exon 6 of ATG5 was used (Fig. 3a). Sequence-specific DNA mutation was confirmed with a sequencing of genomic DNA isolated from the mock (HeLa-sgGFP) and ATG5-knockout (HeLa-sgATG5) cell lines. To test whether ATG5 gene disruption leads to autophagy inhibition, we incubated the mock and ATG5-knockout cell lines with chloroquine, an established inducer of autophagosome accumulation. Fig. 3b demonstrates that chloroquine led to a substantial accumulation of lipidated form of LC3 in HeLa-sgGFP cells. Conversely, LC3-II was undetectable in HeLa-sgATG5 cells and there was a pronounced accumulation of LC3-I, that suggest a compensatory mechanism resulting from disruption of the initial steps of autophagy. Moreover, no autophagosomes could be detected by immunofluorescence microscopy in HeLa-sgATG5 cells, while the autophagosomes were clearly detectable in HeLa-sgGFP cells upon chloroquine incubation (Fig. 3c). These experiments confirm that ATG5 knockout results in a complete autophagy inhibition, and validated this approach as effective for further functional studies with PDT.

### Abolition of ATG5-dependent autophagy sensitizes cells to PDT.

**Fig. 4:**
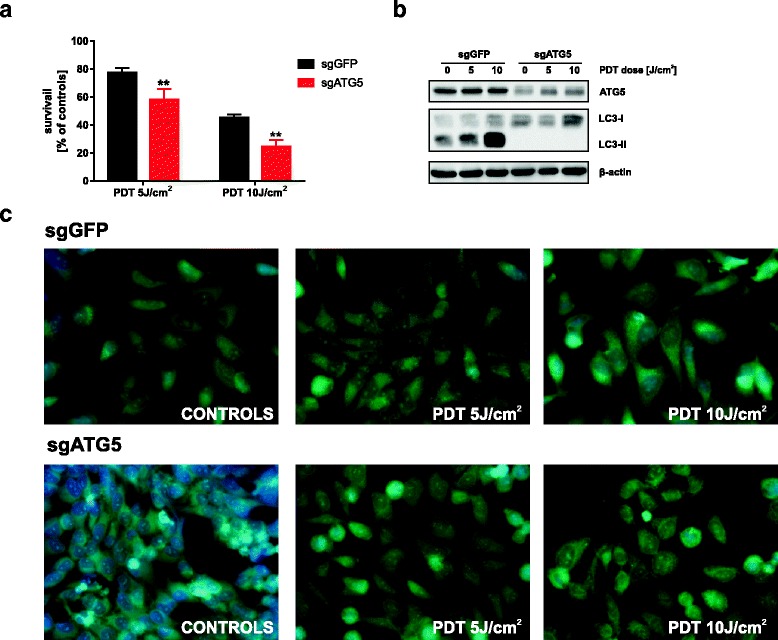
Elimination of ATG5-dependent autophagy sensitizes cells to PDT. **a** HeLa-sgGFP and HeLa-sgATG cells were subjected to in vitro PDT and the cell survival was determined by crystal violet staining 24 h after treatment. The viability in each experimental group is calculated versus its own untreated control. Data represent the mean values of 2 independent experiments ± S.D. (represented by error bars), **p < 0.001 (Student’s *t*-test). **b** To confirm impaired autophagic flux, cells were treated as in (a) and the whole-cell lysates were collected and ATG5 and LC3 levels were analyzed by Western blotting. **c** Immunofluorescence microscopy was used to confirm abrogated autophagosome formation after PDT

To further delineate the role of autophagy in Photofrin-based PDT, we used the ATG5 knockout HeLa cells. HeLa-sgGFP and HeLa-sgATG5 cells were subjected to in vitro PDT, and 24 h post illumination the cytostatic/cytotoxic effects were assessed by crystal violet staining. We found that ATG5 knockout renders cells more sensitive to PDT. As shown in Fig. 4a, this effect could be observed at both light fluencies. As expected, PDT triggered LC3-II accumulation only in HeLa-sgGFP cells (Fig. 4b). Likewise, autophagosomes formation was visualized only in mock, but not in ATG5 knockout cells (Fig. 4c).

### Abrogation of ATG5-dependent autophagy potentiates apoptosis.

**Fig. 5:**
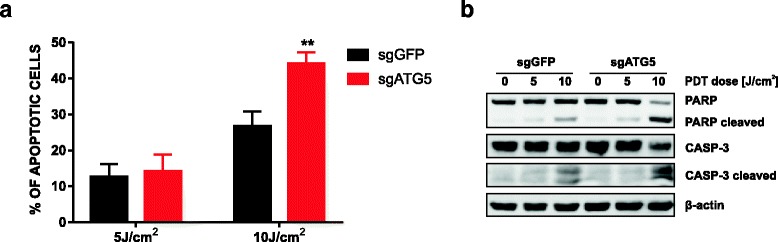
Abrogation of ATG5-dependent autophagy potentiates apoptosis. HeLa-sgGFP and HeLa sgATG cells were subjected to PDT. Cells were collected 24 h after illumination and stained with Annexin V-FITC and propidium iodide. Subsequently, early and late apoptotic cells (annexin V-positive and annexin V as well as propidium iodide double positive, respectively) were quantified by flow cytometry. Data represent the mean values of 2 independent experiments ± S.D. (represented by error bars), **p < 0.001 (Student’s *t*-test). **a** The bars show mean value of annexin V or propidium iodide positive cells. **b** cells were treated as in (a) and whole-cell lysates were collected 24 h after PDT, and the levels of PARP and caspase-3 and their activated cleaved forms were analyzed by Western blotting

It is well documented that both autophagy and apoptosis are involved in determining the cell fate after PDT and that there is a crosstalk between these pathways. To test whether the increased PDT sensitivity of ATG5-knockout cell line results from enhanced apoptosis, we evaluated various markers of apoptotic cell death. Firstly, we examined apoptotic pathway by annexin V/PI staining 24 h post PDT. The number of apoptotic cells after high-dose PDT is significantly higher in HeLa-sgATG5 compared to HeLa-sgGFP cells (Fig. 5a). These results are in agreement with immunoblotting analysis of caspase-3 and PARP cleavage. As shown in Fig. 5b, the amounts of cleaved PARP and cleaved caspase-3 were significantly higher in HeLa-sgATG5 cells under high-dose PDT, which further confirms that ATG5 knockout results in enhanced apoptotic response after PDT at high light fluencies.

### Abrogation of ATG5-dependent autophagy increases protein carbonylation.

**Fig. 6:**
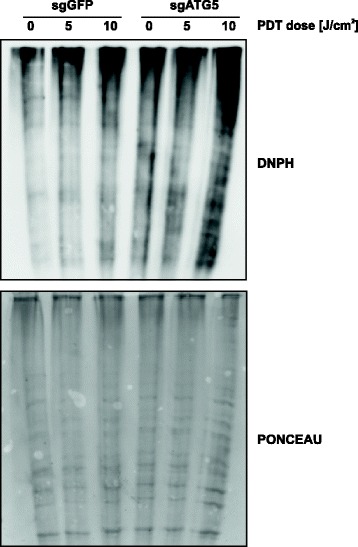
Abrogation of ATG5-dependent autophagy increases protein carbonylation. HeLa-sgGFP and HeLa-sgATG cells were subjected to PDT. Total cell lysates were prepared from tumor cells 24 h after illumination and protein carbonylation was assessed by the 2,4-dinitrophenylhydrazine (DNPH) method (upper panel). To ensure equal protein loading, the blot was stained with Ponceau (lower panel)

Autophagy facilitates removal of oxidatively damaged macromolecules, such as carbonylated proteins. Therefore, we decided to evaluate whether ATG5 knockout affects protein carbonylation after photodynamic therapy. For this purpose, HeLa-sgGFP and HeLa-sgATG5 cells were photo-irradiated and 24 h after illumination, protein carbonylation was evaluated by DNPH method. We found that the amount of carbonylated proteins is significantly higher in ATG5-deficient compared to mock cells, which is further increased upon PDT treatment, in particular at high light fluencies (Fig. 6).

## Discussion

There are numerous studies showing induction of autophagy following photodynamic therapy with different photosensitizers. To our knowledge, so far there has been published only one report addressing the influence of Photofrin on cellular autophagy. The study shows that Photofrin alone, without light exposure, inhibits initial steps of autophagy [23]. However, we observed a robust autophagy induction in our experimental model, which was confirmed by LC3 processing by immunoblotting (Fig. 1a) and detection of autophagic puncta by immunofluorescence (Fig. 1c). Some of the photosensitizers, primarily those accumulating in the lysosomal membranes, were described to cause photodamage to lysosomes, thereby leading to accumulation of autophagosomes due to inhibition of autophagic flux [31, 32]. To address this, we performed an LC3 turnover assay [27]. Increased processing of LC3 after chloroquine co-incubation revealed that Photofrin-PDT leads to stimulation of autophagy rather than inhibition of autophagosomes degradation (Fig. 1b).

To address the role of autophagy in regulating the sensitivity of cancer cells to Photofrin-PDT we downregulated ATG5, a key gene to trigger autophagy, via two different methods: shRNA and CRISPR/Cas9. We found that shRNA-mediated knockdown of ATG5 only marginally affects the sensitivity of cancer cells towards PDT (Fig. 2a). This may be caused by the fact that expression of ATG5 is diminished, but not fully blocked (Fig. 2b). Indeed, we observed moderate autophagy induction in both HeLa and MCF-7 cell lines expressing ATG5-specific shRNA (Fig. 2a). It is possible that minimal expression of autophagy proteins is sufficient for effective autophagy execution, thus PDT efficacy is minimally affected. This observation is in agreement with literature data suggesting that ATG5 downregulation with shRNA is transient and provides incomplete inhibition of autophagy [30]. Thus, we aimed to create a cell line with completely abolished autophagy. For this purpose, we used the CRISPR/Cas9 technique, which leads to the permanent genome editing [33, 34]. We generated a HeLa cell line with genomic disruption of ATG5, rendering abnormal expression of ATG5 protein. In this cell line, autophagy could not be induced by chloroquine, as evidenced by lack of LC3 conversion (Fig. 3b) and no autophagosome formation (Fig. 3c). Furthermore, autophagy was not induced by PDT (Fig. 4b, c). Importantly, we found that ATG5 knockout results in increased sensitivity of HeLa cells to photodynamic therapy (Fig. 4a).

Several lines of evidence suggest that there is a dynamic interplay between autophagy and apoptosis following PDT treatment [20, 25, 35, 36]. It has been described that autophagic response has different roles in apoptosis-deficient and apoptosis-competent cells. The literature data suggest that autophagy serves as a pro-death pathway in cells with impaired apoptosis [35]. The results of our study revealed that PDT-induced apoptosis is more pronounced in autophagy-deficient compared to autophagy-competent cells (Fig. 5).

It is widely accepted that PDT leads to accumulation of oxidatively damaged proteins [24, 37]. Moreover, it is believed that autophagy is one of the crucial mechanisms of removal of oxidatively damaged macromolecules and organelles, contributing to cellular homeostasis in oxidatively stressed cells [38]. We have previously reported that PDT triggers a buildup of ubiquitinated and carbonylated proteins, and that PDT cytotoxicity can be potentiated by proteasome inhibition [24]. Here, we report that blocking another arm of the protein degradation system, i.e. autophagy [39], contributes to enhanced accumulation of carbonylated proteins (Fig. 6), as well as increased sensitivity towards PDT (Figs. 4 and 5).

### Conclusions

Altogether, our results indicate that inhibition of autophagy via CRISPR/Cas9-mediated genomic disruption of ATG5, enhances cytotoxicity of Photofrin-based photodynamic therapy in HeLa cells. In the autophagy-deficient cells PDT triggers enhanced apoptotic response. Moreover, we demonstrate that autophagy is involved in recycling of carbonylated proteins, which accumulate in response to PDT. Further studies are needed to investigate the role of autophagy in response to PDT in different cancer cell types and to conclude about possible clinical implications of these findings.

## Abbreviations

ATG5: Autophagy related 5
CQ: Chloroquine
CRISPR/Cas9: Clustered Regularly Interspaced Short Palindromic Repeats/CRISPR-associated protein-9 nuclease
DNPH: 2,4-Dinitrophenylhydrazine
GFP: Green fluorescent protein
LC3: Microtubule-associated protein 1A/1B–light chain 3
PAM: Protospacer adjacent motif
PARP: Poly (ADP-ribose) polymerase
PDT: Photodynamic therapy
PI: Propidium iodide
S.D.: Standard deviation
shRNA: Short hairpin RNA
